# Pre-Existing Anxiety and Depression in Injured Older Adults: An Under-Recognized Comorbidity With Major Health Implications

**DOI:** 10.1097/AS9.0000000000000217

**Published:** 2022-12-07

**Authors:** Damaris Ortiz, Anthony J. Perkins, Mikita Fuchita, Sujuan Gao, Emma Holler, Ashley D. Meagher, Sanjay Mohanty, Dustin D. French, Sue Lasiter, Babar Khan, Malaz Boustani, Ben Zarzaur

**Affiliations:** From the * Department of Surgery, Indiana University School of Medicine, Indianapolis, IN; † Sidney and Lois Eskenazi Hospital Smith Level One Trauma Center, Indianapolis, IN; ‡ Center of Health Innovation and Implementation Science, Center for Translational Science and Innovation, Indianapolis, IN; § Department of Biostatistics and Health Data Science, Indiana University School of Medicine, Indianapolis, IN; ∥ Division of Critical Care, Department of Anesthesiology, University of Colorado Denver, Anschutz Medical Campus, Aurora, CO; ¶ Department of Epidemiology and Biostatistics, Indiana University, Bloomington, IN; # Indiana University Health, Methodist Hospital Level One Trauma Center, Indianapolis, IN; ** Department of Ophthalmology, Feinberg School of Medicine, Northwestern University, Chicago, IL; †† Department of Medical Social Sciences, Feinberg School of Medicine, Northwestern University, Chicago, IL; ‡‡ Center for Health Services and Outcomes Research, Feinberg School of Medicine, Northwestern University, Chicago, IL; §§ Veterans Affairs Health Services Research and Development Service, Chicago, IL; ∥∥ School of Nursing and Health Studies, Health Sciences District, University of Missouri, Kansas City, MO; ¶¶ Department of Medicine, Indiana University School of Medicine, Indianapolis, IN; ## Department of Surgery, University of Wisconsin School of Medicine, Madison, WI.

**Keywords:** anxiety, depression, injured, older adult, psychiatric comorbidity, trauma

## Abstract

To compare differences in baseline depression and anxiety screenings between older injured patients with pre-existing diagnoses and those without.

**Background:**

Little is known about the prevalence and impact of psychiatric comorbidities on early postinjury depression and anxiety in nonneurologically injured older adults.

**Methods:**

This was a retrospective post-hoc analysis of data from the Trauma Medical Home, a multicenter randomized controlled trial (R01AG052493-01A1) that explored the effect of a collaborative care model on postinjury recovery for older adults compared to usual care.

**Results:**

Nearly half of the patients screened positive for at least mild depressive symptoms as measured by the Patient Health Questionnaire-9. Forty-one percent of the patients screened positive for at least mild anxiety symptoms as measured by the Generalized Anxiety Disorder Scale. Female patients with a history of concurrent anxiety and depression, greater injury severity scores, and higher Charlson scores were more likely to have mild anxiety at baseline assessment. Patients with a history of depression only, a prior history of depression and concurrent anxiety, and higher Charlson scores (greater medical comorbidity) had greater odds of at least mild depression at the time of hospital discharge after traumatic injury.

**Conclusions:**

Anxiety and depression are prevalent in the older adult trauma population, and affect women disproportionately. A dual diagnosis of depression and anxiety is particularly morbid. Mental illness must be considered and addressed with the same importance as other medical diagnoses in patients with injuries.

## Abstract 2

Mini-abstract: We evaluated injured older adults and compared baseline anxiety and depression screenings between those with and without a pre-existing diagnosis. Forty to fifty percent of the patients screened positive, and one-quarter of these patients did not have a pre-existing history. Medical comorbidity and prior dual diagnosis of depression and anxiety were most associated with increased anxiety and depression symptoms.

## INTRODUCTION

Recovery from injury is complicated by chronic medical illness and psychiatric comorbidity in older adults.^1–3^ In nontrauma populations, co-existing psychiatric and medical conditions have been associated with adverse outcomes. For example, higher severity of depression symptoms is associated with increased mortality when controlling for medical comorbidities.^4^ Likewise, a concurrent chronic medical illness decreases quality of life and physical functioning in community-dwelling older adults with depression.^5^ Postinjury quality of life and physical functioning are influenced by biopsychosocial factors,^6,7^ and psychiatric comorbidity plays an important role in recovery after traumatic injury. However, epidemiological studies have suggested that pre-existing psychiatric comorbidities are likely to be under-diagnosed in older patients.^8,9^

Primary care literature describes the challenges of identifying and treating patients with co-occurring mental and medical illnesses.^11^ However, such discussion has been limited in the trauma literature.^12^ Previous studies of injured patients suggest a high prevalence of psychiatric comorbidity in the injured older adult population.^13,14^ While some studies describe outcomes in trauma patients with pre-existing mental illness,^13–16^ the literature is largely centered around depression and posttraumatic stress disorder (PTSD) as sequelae of injury.^17–22^ Recent investigations have considered the potential effects of pre-existing psychiatric comorbidity, especially PTSD and depression on trauma patients.^23,24^ The latest edition of the American College of Surgeons Committee on Trauma Resources for the Injured Patient recommends that all trauma centers “have a protocol in place to screen and refer patients at high risk for mental health issues following injury” (Standard 5.29).^25^ Screening is the first step in identifying at-risk individuals and providing the services they need.^26^ Automated screening of trauma patients at high risk for the development of PTSD has been described.^27^ This article reviewed multiple candidate predictors and included 10 in the final model. Prior history of PTSD by ICD-9 code as well as prior history of any other ICD-9 psychiatric diagnosis were included. This suggests the importance of pre-existing psychiatric comorbidity on psychological outcomes of the injured patient, a subject which has been relatively unstudied in older adults. Depression and anxiety are the most common psychiatric diagnoses affecting the older adult^9,10^ and warrant further study.

The prevalence of psychiatric comorbidities and their impact on recovery after traumatic injuries in older adults remains unclear. This study aimed to describe the prevalence of pre-existing depression and anxiety in injured older adults and determine their impact on postinjury depression and anxiety symptoms.

## METHODS

### Study Setting and Population

This was a post-hoc analysis of data from Trauma Medical Home, a multicenter randomized controlled trial (R01AG052493-01A1) that explored the effect of a collaborative care model on postinjury recovery for older adults compared to usual care. Patients were recruited from four level-1 trauma centers in the Midwest. This study was approved by the Indiana University Institutional Review Board and followed the ethical principles of the Declaration of Helsinki.

### Inclusion and Exclusion Criteria

This study included injured adults aged ≥50 years with an injury severity score (ISS) of ≥9 who resided within 50 miles of the admitting trauma center and had telephone access. Patients with significant head injury, neurodegenerative disease, stroke, spinal cord injury, burns, pregnancy, advanced malignancy, alcohol or drug use disorder within the previous 6 months, or sensory impairment that precluded study participation were excluded. Alcohol or drug use disorder was determined by chart review, a positive drug screen on admission, a blood ethanol level 250 mg/dL or above, or patients involved in motor vehicle crashes with a blood ethanol level of >80 mg/dL. The detailed study protocol has been previously published.^28^ Informed consent was obtained from all participants before study enrollment. At the time of enrollment, all participants completed baseline assessments which included depression screening using the Patient Health Questionnaire-9 (PHQ-9) and anxiety screening using the Generalized Anxiety Disorder Scale (GAD-7). Enrollment occurred at the time of hospital discharge. Participants were then randomized to either usual care or the Trauma Medical Home (TMH) intervention.

### Exposure Variables

The exposure variables of interest were history of pre-existing depression and anxiety. This history was determined retrospectively using any prior ICD-10 codes and medication history in the electronic medical record. A list of the common anxiety and depression medications reviewed is available in a Supplemental file, http://links.lww.com/AOSO/A178.

### Outcomes and Measures

The primary outcome was the presence of symptoms of anxiety (GAD-7 score ≥ 5) and depression (PHQ-9 score ≥ 5) at the time of hospital discharge. The PHQ-9 is a nine-item depression scale with a total score ranging from 0 to 27, and the GAD-7 is a seven-item anxiety scale with a total score from 0 to 21. Scores of 5, 10, and 15 represent the cutoff points for mild, moderate, and severe symptoms, respectively, for both scales. Both are derived from the PHQ and have good internal consistency and validity for the diagnosis of major depression and generalized anxiety disorders.^29,30^

### Covariates

Demographics, including sex, insurance type, and education, were collected from the medical record at the time of enrollment. Data on self-identified race, income, and marital status were obtained from a patient questionnaire. Injury characteristics, including injury mechanism and ISS, were obtained from chart review, and the ISS was calculated based on the injuries listed in the problem list. Medical comorbidity was represented by the Charlson comorbidity score, and this information was obtained from the electronic medical record.

### Statistical Approach

Chi-square tests and Wilcoxon rank sum tests were used to compare whether patient characteristics differed according to pre-existing history of anxiety/depression. We also used chi-square tests and Wilcoxon rank sum tests to compare whether patient characteristics differed according to mild depression and anxiety during the baseline visit. Two logistic regression analyses were performed to evaluate the relationship between prior history of anxiety and/or depression and the presence of mild or more severe symptoms at the time of hospital discharge: (1) mild depression (PHQ-9 score of 5 or greater) and (2) mild anxiety (GAD-7 score of 5 or greater). A sensitivity analysis was then performed with higher symptom severity cutoffs: moderate depression (PHQ-9 score ≥ 10), and moderate anxiety (GAD-7 score ≥ 10). All models were controlled for age, sex, race, marital status, medical comorbidities, and injury characteristics, which are potential confounders. Participants with missing data were excluded from the analysis. Statistical significance was set at *P* <0.05, and all analyses were performed using SAS 9.4 (version 9.4; Cary, NC). The STROBE guidelines were used to ensure proper reporting of methods, results, and discussion.

## RESULTS

### Cohort Characteristics

In total, 448 patients were enrolled in this study. Eighteen patients were enrolled but not randomized due to withdrawal from the study or incomplete baseline assessments. Randomization was performed after baseline assessments were completed. Of the 430 patients randomized in the study, 33 were excluded due to incomplete prior history of depression or anxiety, or incomplete baseline GAD-7 and/or PHQ-9. In total, 397 patients were included in the analysis. The median time from admission to assessment was 4 days with a range from 0 to 58 days. Table 1 and Supplemental Table 1, http://links.lww.com/AOSO/A179, summarize patient demographics and medical characteristics based on depression and anxiety history. Most patients were White, female, married, and had an average age of 69 (SD 10.5) years. Approximately one-third of the patients had a history of either depression or anxiety (35.3%), 16.6% had a history of depression only, 4.5% had a history of anxiety only, and 14.1% had a history of both depression *and* anxiety. The patient groups differed in terms of sex, marital status, injury characteristics, and median Charlson comorbidity score.

**TABLE 1.** Demographics and Medical Conditions by Depression/Anxiety History in EMR

	Overall(n = 397)	No Depression or Anxiety(n = 257)	Depression Only(n = 66)	Anxiety Only(n = 18)	Depression and Anxiety(n = 56)	*P*
Age, y						0.637
50–64	146 (36.8)	103 (40.1)	21 (31.8)	5 (27.8)	17 (30.4)	
65–79	184 (46.3)	110 (42.8)	34 (51.5)	10 (55.6)	30 (53.6)	
80+	67 (16.9)	44 (17.1)	11 (16.7)	3 (16.7)	9 (16.1)	
Sex						<0.001
Female	212 (53.4)	111 (43.2)	47 (71.2)	13 (72.2)	41 (73.2)	
Male	185 (46.6)	146 (56.8)	19 (28.8)	5 (27.8)	15 (26.8)	
Race						0.633
African-American	33 (8.3)	25 (9.8)	4 (6.1)	2 (11.1)	2 (3.6)	
Other	3 (1.8)	2 (0.8)	0 (0.0)	0 (0.0)	1 (1.8)	
White	359 (90.0)	229 (89.5)	62 (93.9)	16 (88.9)	52 (94.5)	
Insurance type						0.190
Private	114 (28.8)	81 (31.6)	17 (25.8)	4 (22.2)	12 (21.4)	
Medicare	103 (26.0)	67 (26.2)	15 (22.7)	4 (22.2)	17 (30.4)	
Medicaid	7 (1.8)	2 (0.8)	3 (4.6)	0 (0.0)	2 (3.6)	
Medicare + Medicaid	12 (3.0)	4 (1.6)	4 (6.1)	2 (11.1)	2 (3.6)	
Medicare + Private	125 (31.6)	75 (29.3)	22 (33.3)	6 (33.3)	22 (39.3)	
None	11 (2.8)	9 (3.5)	1 (1.5)	1 (5.6)	0 (0.0)	
Other (+gvt)	8 (4.0)	18 (7.0)	4 (6.1)	1 (5.6)	1 (1.8)	
Injury characteristics						0.011
Fall	217 (54.7)	124 (48.2)	46 (69.7)	11 (61.1)	36 (64.3)	
Motor vehicle accident	126 (31.7)	98 (38.1)	13 (19.7)	3 (16.7)	12 (21.4)	
Other	54 (13.6)	35 (13.6)	7 (10.6)	4 (22.2)	8 (14.3)	
Median ISS	10 (9–14)	10 (9–14)	10 (9–14)	11 (9–14)	10 (9–14)	0.407
Median Charlson	0 (0–1)	0 (0–1)	1 (0–2)	0 (0–1)	1 (0–2.5)	0.004

### In-Hospital Depression and Anxiety Screening

Nearly half of the patients screened positive for at least mild depressive symptoms in the hospital, as measured by the PHQ-9. In the univariate analysis, patients with a history of prior anxiety and/or depression were more likely to screen positive. Forty-one percent of patients screened positive for mild anxiety symptoms at the hospital. Patients who screened positive for anxiety were more likely to be female, have a prior history of depression and/or anxiety, and have more comorbid medical conditions, as shown in Table 2. Forty-eight of 203 (23.6%) patients that had no depressive symptoms at baseline had a prior history of depression or anxiety in the EMR; 63 of 235 (26.8%) patients who had no anxiety symptoms at baseline had a prior history of depression or anxiety in the EMR; 102 of 194 (52.6%) patients with depressive symptoms had no recorded prior history of anxiety or depression; and 85 of 162 patients (52.5%) patients with baseline anxiety symptoms had no recorded prior history of anxiety or depression. These results are presented in Table 2. Additional demographic and medical characteristics by anxiety and depression symptom screening are shown in Supplemental Table 2, http://links.lww.com/AOSO/A179.

**TABLE 2.** Demographics and Medical Characteristics by Depression/Anxiety Symptoms (n = 397)

	No Depression (n = 203)	Depression (PHQ-9 ≥ 5) (n = 194)	*P*	No Anxiety (n = 235)	Anxiety (GAD-7 ≥ 5) (n = 162)	*P*
Age, y			0.838			0.222
50–64	73 (36.0)	73 (37.6)		83 (35.3)	63 (38.9)	
65–79	97 (47.8)	87 (44.8)		106 (45.1)	78 (48.1)	
80+	33 (16.3)	34 (17.5)		46 (19.6)	21 (13.0)	
Sex			0.058			0.019
Female	99 (48.8)	113 (58.2)		114 (48.5)	98 (60.5)	
Male	104 (51.2)	81 (41.8)		121 (51.5)	64 (39.5)	
Race			0.863			0.948
African-American	17 (8.4)	16 (8.3)		19 (8.1)	14 (8.7)	
Hispanic	2 (1.0)	1 (0.5)		2 (0.9)	1 (0.6)	
White	183 (90.6)	176 (91.2)		213 (91.0)	146 (90.7)	
Injury characteristics			0.772			0.568
Fall	108 (53.2)	109 (56.2)		131 (55.7)	86 (53.1)	
Motor vehicle accident	65 (32.0)	61 (31.4)		70 (29.8)	56 (34.6)	
Other	30 (14.8)	24 (12.4)		34 (14.5)	20 (12.3)	
Prior depression, anxiety			<0.001			<0.001
None	155 (76.4)	102 (52.6)		172 (73.2)	85 (52.5)	
Depression only	23 (11.3)	43 (22.2)		37 (15.7)	29 (17.9)	
Anxiety only	7 (3.4)	11 (5.7)		9 (3.8)	9 (5.6)	
Depression and anxiety	18 (8.9)	38 (19.6)		17 (7.2)	39 (24.1)	
Median ISS	10 (9–14)	10 (9–14)	0.620	10 (9–14)	10 (9–14)	0.105
Median Charlson	0 (0-1)	1 (0-2)	0.001	0 (0-1)	1 (0-2)	0.023

### Regression Analyses: Mild Depression and Anxiety

In model 1, we examined factors associated with mild or greater depression symptoms (PHQ-9 score ≥ 5) at discharge. We found that patients with a history of depression only [odds ratio (OR), 2.62; 95% confidence interval (95% CI), 1.44–4.79], prior history of both depression and anxiety [OR (95% CI), 2.76 (1.42–5.34)], and higher Charlson scores [OR (95% CI), 1.28 (1.08–1.51)] had greater odds of at least mild depression at the time of hospital discharge after traumatic injury. In model 2, we examined factors associated with at least mild anxiety (GAD-7 score ≥ 5) at discharge. We found that female patients [OR (95% CI), 1.77 (1.08–2.88)], with a history of both anxiety and depression [OR (95% CI), 4.72 (2.36–9.46)], greater ISS [OR (95% CI), 1.07 (1.01–1.12)], and a higher Charlson score [OR (95% CI), 1.21 (1.02–1.42)] were more likely to have at least mild anxiety. These results are presented in Table 3.

**TABLE 3.** Logistic Regression Results (Mild Depression or Anxiety)

	Depression (PHQ-9 ≥ 5)		Anxiety (GAD-7 ≥ 5)	
	OR (95% CI)	*P*	OR (95% CI)	*P*
Age, y		0.388		0.299
50–64 (reference)	1.00		1.00	
65–79	0.71 (0.43, 1.17)	0.176	0.83 (0.50, 1.39)	0.476
80+	0.86 (0.44, 1.68)	0.663	0.57 (0.28, 1.16)	0.121
Female	1.13 (0.71, 1.80)	0.604	1.77 (1.08, 2.88)	0.023
Race		0.749		0.528
African-American	1.05 (0.49, 2.28)	0.894	1.55 (0.69, 3.44)	0.286
Other	0.38 (0.03, 4.99)	0.458	0.65 (0.05, 8.55)	0.743
White (reference)	1.00		1.00	
Married	0.93 (0.60, 1.46)	0.757	2.07 (1.28, 3.35)	0.003
Injury characteristics		0.893		0.716
Fall	1.15 (0.58, 2.29)	0.696	1.12 (0.54, 2.33)	0.753
Motor vehicle accident	1.19 (0.58, 2.41)	0.640	1.33 (0.63, 2.80)	0.456
Other (reference)	1.00		1.00	
Prior depression, anxiety		0.001		<0.001
None (reference)	1.00		1.00	
Depression only	2.62 (1.44, 4.79)	0.002	1.63 (0.89, 2.97)	0.115
Anxiety only	2.64 (0.96, 7.30)	0.061	1.88 (0.68, 5.17)	0.224
Depression and anxiety	2.76 (1.42, 5.34)	0.003	4.72 (2.36, 9.46)	<0.001
ISS	1.05 (1.00, 1.10)	0.076	1.07 (1.01, 1.12)	0.019
Charlson	1.28 (1.08, 1.51)	0.005	1.21 (1.02, 1.42)	0.027

### Regression Analyses: Moderate-to-Severe Depression and Anxiety

In model 3, we examined factors associated with moderate-to-severe depression (PHQ-9 score ≥ 10) at discharge. We found that patients who were injured in a motor vehicle accident [OR (95% CI),3.01 (1.03–8.78)], had a history of anxiety [OR (95% CI), 3.23 (1.01–10.32)], a prior history of both depression and anxiety [OR (95% CI), 4.60 (2.22–9.56)], and a higher Charlson score [OR (95% CI), 1.41 (1.18–1.68)] had significantly greater odds of having moderate-to-severe depression. In model 4, we examined variables associated with moderate-to-severe anxiety (GAD-7 score ≥ 10) at discharge. Patients with a history of both depression and anxiety [OR (95% CI), 3.76 (1.75–8.06)], and those with a higher Charlson score were more likely to have moderate-to-severe anxiety [OR (95% CI), 1.41 (1.17–1.70)]. These results are presented in Table 4.

**TABLE 4.** Logistic Regression Results (Moderate Depression or Anxiety)

	Depression (PHQ-9 ≥ 10)		Anxiety (GAD ≥ 10)	
	OR (95% CI)	*P*	OR (95% CI)	*P*
Age, y		0.443		0.153
50–64 (reference)	1.00		1.00	
65–79	0.76 (0.41, 1.44)	0.406	0.50 (0.25, 1.04)	0.063
80+	0.56 (0.23, 1.39)	0.211	0.51 (0.20, 1.35)	0.177
Female	0.99 (0.54, 1.82)	0.969	1.86 (0.92, 3.76)	0.084
Race		0.794		0.657
African-American	1.29 (0.50, 3.34)	0.605	1.49 (0.54, 4.13)	0.444
Other	1.92 (0.13, 29.58)	0.639	2.15 (0.14, 32.51)	0.581
White (reference)	1.00		1.00	
Married	0.76 (0.43, 1.34)	0.342	1.02 (0.53, 1.95)	0.954
Injury characteristics		0.130		0.925
Fall	2.32 (0.80, 6.68)	0.120	1.01 (0.36, 2.84)	0.981
Motor vehicle accident	3.01 (1.03, 8.78)	0.044	1.17 (0.41, 3.37)	0.770
Other (reference)	1.00		1.00	
Prior depression, anxiety		<0.001		0.003
None (reference)	1.00		1.00	
Depression only	1.30 (0.60, 2.83)	0.508	0.89 (0.36, 2.23)	0.801
Anxiety only	3.23 (1.01, 10.32)	0.049	1.08 (0.22, 5.22)	0.921
Depression and anxiety	4.60 (2.22, 9.56)	<0.001	3.76 (1.75, 8.06)	0.001
ISS	1.05 (0.99, 1.12)	0.091	1.01 (0.95, 1.09)	0.713
Charlson	1.41 (1.18, 1.68)	<0.001	1.41 (1.17, 1.70)	<0.001

### PTSD Screening

Only patients randomized to the TMH intervention were screened for PTSD with the PTSD checklist—Civilian Version (PCL-C). Those with mild anxiety on GAD-7 had significantly (*P* = 0.001) higher PCL-C scores (mean = 24.8, SD = 9.4) than those without mild anxiety (mean = 20.7, SD = 5.2).

## DISCUSSION

In this retrospective study, we investigated the prevalence of pre-existing depression and anxiety and their association with depression and anxiety symptoms at hospital discharge among 397 older adults who required hospital admission after traumatic injuries. We found that 35% of our patients had pre-existing depression or anxiety, higher than the lifetime prevalence estimates in the United States (20.8% for mood disorders, 16.6% for major depressive disorder, and 28.8% for anxiety disorders).^31^ Pre-existing diagnosis of both depression with anxiety and a higher Charlson comorbidity score were significantly associated with depression and anxiety symptoms at hospital discharge. These relationships persisted in our sensitivity analysis using a higher cutoff for depression and anxiety symptom severity.

Unsurprisingly, the Charlson comorbidity score was strongly associated with more depression and anxiety symptoms, consistent with previous reports. Some nontrauma literature describes the interplay between medical and psychiatric comorbidity, as well as the negative psychological effects of chronic illness, although this is an area that needs further study.^2,32,33^ In one study, patients with chronic illness with comorbid anxiety or depression reported significantly more medical symptoms than those with chronic illness alone.^34^ In another study, depressed patients with medical comorbidities reported significantly lower quality of life and were at higher risk for disability than those without depression.^5^ These findings suggest that older patients with pre-existing medical comorbidities and anxiety or depression are already at high risk for negative psychological outcomes.^35,36^ This is an underappreciated area of study in the trauma literature despite the importance of mood symptoms on outcomes after injury.

One of the key findings of our study was that 14% of the cohort was previously diagnosed with both depression *and* anxiety. This is significantly higher than the 12-month national prevalence of 3% of co-occurring mood and anxiety disorders among older adults reported in 2010.^10^ The distinction between the effects of anxiety or depression existing in isolation versus existing as concurrent conditions is important. Comorbidity is common among anxiety and depression disorders. Up to half of all patients with depression have significant anxiety and 60%–90% of older adults with anxiety also have depression.^37,38^ In the outpatient setting, concurrent diagnosis of depression and anxiety was associated with a decreased likelihood of remission, worse symptoms, more severe depression, and increased suicidality.^39^ Anxious depression is also associated with increased healthcare utilization and often requires difficult long-term treatment. Despite its high prevalence, it often goes undiagnosed.^38–40^ Identification of comorbid anxiety and depression in addition to traumatic injuries may require specialized care, including pharmacological treatment and a focus on psychiatric symptom management. It is evident that the role of psychiatric comorbidity is as critical as that of chronic medical illnesses in patients’ overall health. This is also likely true during recovery after injury, as shown in prior investigations. A national United States study demonstrated the impact of postinjury depression and PTSD on functional decline in the first year after injury.^18^ This study cohort aged 18–84 years had a 14% prevalence of preinjury depression. A stepped care intervention trial targeting PTSD symptoms in a cohort 18 years and older accounted for preinjury PTSD symptoms which were present in 62.8% of participants.^23^ In a similar study utilizing Collaborative Care Models (CCM) to reduce PTSD symptoms, 34.5% of the cohort had preinjury PTSD symptoms.^24^ Preinjury anxiety and its overlap with pre-existing depression remains largely unexamined in this population.

It is unclear why the positive screening rates with the GAD-7 and PHQ-9 were higher than the pre-existing prevalence of depression and anxiety. This could be because the traumatic event itself caused an increase in depression and anxiety symptoms.^19^ The onset of symptoms may have occurred early in the hospital course. Depression and PTSD affect up to 6% and 20% of patients in the first year after hospitalization for injury, respectively.^20,21^ Symptoms of acute stress disorder present within a month of injury, and are often a precursor to PTSD.^22^ Its presentation includes anxiety symptoms as well as symptoms that overlap with depression.^22^ This may have contributed to the high prevalence of anxiety and depression symptoms at the baseline assessment. Alternatively, patients with undiagnosed depression and anxiety may have been identified during the screening process. In either instance, screening provides an opportunity for secondary prevention of anxiety and depression: early detection and intervention to avoid symptom progression.^41^ Early work has focused on the secondary prevention of PTSD in trauma survivors^42^ and primary prevention of depression in adults with chronic medical illness.^43^ This is an area that requires further study.

While anxiety and mood disorders decline with age, they remain prevalent, especially in women.^9,35,36^ We found that more than 70% of the patients with pre-existing depression or anxiety were women. Women comprised 60% of those with depression or anxiety symptoms at baseline assessment. The reasons for the higher prevalence of depression and anxiety in women are likely multifactorial and require further investigation, given the significant burden of disease.^44^ Despite the higher prevalence in women, there is currently no difference in depression and anxiety treatments based on sex. Given the complexity of psychiatric illness and its interplay with chronic medical conditions, treatment models should be able to address a wide range of individual contributing factors.

One such example is the CCM, a primary care-based model that integrates behavioral and medical health care. CCM utilizes individualized treatment protocols that can be adjusted as needed based on measured assessments, and has the potential to be applied to multiple patient populations, not just in the primary care setting. The IMPACT study (Improving Mood-Promoting Access to Collaborative Treatment) is the largest randomized controlled trial to date, utilizing this model for the treatment of depression in adults aged 60 years or older in the primary care setting. Patients in the intervention arm had a 50% or greater reduction in depressive symptoms from baseline compared with 19% of patients receiving usual care.^45^ A similar treatment response was reported in patients with comorbid medical illness.^46^ Recent collaborative care interventions have been studied to reduce postinjury PTSD symptoms and found a significant reduction at 6 months, but not at 3- or 12-month follow-up.^24^ Further study of collaborative care interventions for injured patients is needed. Collaborative care provides the opportunity to screen for and identify symptoms of mental illness as well as comorbid medical conditions that contribute to disability and further injury. The results from the application of CCM to trauma care for older injured adults in the Trauma Medical Home trial are pending. New interventions for trauma centers need to differ based on patient demographics and local restrictions and limitations. Similarly, programs tailored to specific populations, such as older adults, must provide the ability to identify individual needs and address them adequately.

### Strengths and Limitations

This study had some important limitations. While this was a multicenter trial, all centers were in the Midwest region of the United States, which may limit generalizability. The study also enrolled a homogeneous population (88.8% non-Hispanic white) which also limits the generalizability of the findings. We did not investigate the effects of treatment for those with pre-existing depression or anxiety. Because this study only evaluated depression and anxiety symptoms at the time of hospital discharge after injury, we were unable to evaluate long-term outcomes. Preinjury history of depression and anxiety was obtained via medical record of ICD-9 and ICD-10 codes and medications which would likely underestimate the true prevalence of these conditions. The validity of the GAD-7 in this patient population may also be limited due to DSM-V criteria stating that “generalized anxiety symptoms are not to be diagnosed if anxiety symptoms are better explained by exposure to a traumatic stressor/posttraumatic stress disorder.”^47^ Only baseline assessments were utilized in this investigation, limiting the GAD-7 and PHQ-9 to tracking of anxiety and depression symptoms, not use as diagnostic tools. Nevertheless, this does not detract from the finding that patients with pre-existing depression and anxiety, and higher Charlson scores were more likely to have anxiety and depression symptoms. This relationship persisted in the sensitivity analysis for moderate-to-severe symptoms for both anxiety and depression.

Despite these limitations, our study is novel in its investigation of the impact of pre-existing depression and anxiety, the most common mental illnesses, on early mood symptoms after injury using multicenter data collected prospectively. We also investigated the combined impact of depression and co-occurring anxiety, which has not been previously described in the literature for patients with traumatic injury. This study is the first to describe the significant prevalence of pre-existing depression and anxiety in nonneurologically injured older adults. Finally, this study provides a context for the importance of identifying and addressing mood symptoms in early recovery after injury.

## CONCLUSIONS

In nonneurologically injured older adults, one-third of the patients had a prior history of either depression or anxiety diagnosis. Pre-existing dual diagnoses of depression and anxiety, and concurrent medical conditions were most strongly associated with depression and anxiety symptoms at the time of discharge after injury. Anxiety and depression are overrepresented in the older adult trauma population compared to the general population and disproportionately affect women. The presence of mental illness must be considered and addressed in the same manner as other medical diagnoses in the injured patient. Future directions include further testing of the CCM in injured patients and designing and testing other interventions for the secondary prevention of depression and anxiety after injury.

## Acknowledgments

This manuscript has been approved for publication by all the authors. All authors contributed significantly to this project’s conception and design, data acquisition, analysis, and interpretation, or manuscript preparation and critical revision. This manuscript has not been published elsewhere, and is not under consideration for publication elsewhere. If accepted, this will not be reproduced elsewhere in the same form.

## Supplementary Material


