# Supplementary material for: Pre-Existing Anxiety and Depression in Injured Older Adults: An Under-Recognized Comorbidity With Major Health Implications
Source: Ann Surg Open. 2022 Dec 7;3(4):e217. doi: 10.1097/AS9.0000000000000217 (PMC9780044; doi:10.1097/AS9.0000000000000217)
Supplement: Supplementary file 1 [file as9-3-e217-s001.pdf]

Pre-Existing Medication Use for TMH Subanalysis

Antidepressants:

Fluoxetine, Sertraline, Citalopram, Paroxetine, Venlafaxine, Duloxetine, Mirtazapine, Bupropion

Source: Indiana University Center for Aging Research

Antianxiety medications:

Xanax, alprazolam, lorazepam, Ativan, buspirone, Lexapro, hydroxyzine, Buspar, valium, diazepam, escitalopram, Cymbalta, Effexor XR, Paxil, duloxetine, venlafaxine, paroxetine, alprazolam intensol, atenolol, Tenormin, tranxene, lorazepam intensol, oxazepam, Paxil CR, prochlorperazine, amitriptyline/perphenazine, chlordiazepoxide, clorazepate, diazepam intensol, doxepin, pexeva, amitriptyline/chlordiazepoxide, compro, corgard, meprobamate, nadolol, tanxene t-tab, trifluoperazine

Source: all non-“off label” and non-“OTC” marked drugs from this list:

https://www.drugs.com/condition/anxiety.html?category_id=&include_rx=true&submitted=true&page_number=2

New prescription = had not had a Rx for this medication for 1 year prior to enrollment and has new Rx for one of these meds after enrollment.
