# Supplementary material for: Pre-Existing Anxiety and Depression in Injured Older Adults: An Under-Recognized Comorbidity With Major Health Implications
Source: Ann Surg Open. 2022 Dec 7;3(4):e217. doi: 10.1097/AS9.0000000000000217 (PMC9780044; doi:10.1097/AS9.0000000000000217)
Supplement: Supplementary file 2 [file as9-3-e217-s002.pdf]

Supplemental Table 1- Additional demographic information by anxiety/depression history

	Overall (n=397)	No Depression or Anxiety (n=257)	Depression Only (n=66)	Anxiety Only (n=18)	Depression and Anxiety (n=56)	P-value
**Income**						0.160
< $25,000	61 (15.7)	34 (13.5)	17 (26.2)	1 (5.6)	9 (16.4)	
$25,000 to < $50,000	72 (18.5)	50 (19.9)	11 (16.9)	2 (11.1)	9 (16.4)	
$50,000 to < $75,000	37 (9.5)	30 (12.0)	2 (3.1)	1 (5.6)	4 (7.3)	
$75,000 or more	72 (18.5)	48 (19.1)	9 (13.8)	6 (33.3)	9 (16.4)	
Unknown	147 (37.8)	89 (35.5)	26 (40.0)	8 (44.4)	24 (43.6)	
**Marital Status**						0.029
Married	224 (56.6)	154 (60.2)	28 (42.4)	13 (72.2)	29 (51.8)	
Divorced/widow/separated/Single	172 (43.4)	102 (39.8)	38 (57.6)	5 (27.8)	27 (48.2)	
**Education**						0.142
High school equiv or less	129 (33.9)	88 (35.8)	23 (36.5)	7 (38.9)	11 (20.4)	
Some college	66 (17.3)	41 (16.7)	8 (12.7)	3 (16.7)	14 (25.9)	
Associate, Bachelor, or vocation	113 (29.7)	69 (28.0)	25 (39.7)	3 (16.7)	16 (29.6)	
Master or Doctorate	73 (19.2)	48 (19.5)	7 (11.1)	5 (27.8)	13 (24.1)	

Abbreviations: gvt=government, equiv=equivalent

Supplemental Table 2: Additional demographic information by anxiety and depression symptoms

	No Depression (n=203)	Depression (PHQ-9≥5) (n=194)	P-value	No Anxiety (n=235)	Anxiety (GAD-7≥5) (n=162)	P-value
**Insurance Type**			0.092			0.236
Private	67 (33.2)	47 (24.2)		70 (29.8)	44 (27.3)	
Medicare	48 (23.8)	55 (28.4)		54 (23.0)	49 (30.4)	
Medicaid	1 (0.5)	6 (3.1)		2 (0.9)	5 (3.1)	
Medicare + Medicaid	3 (1.5)	9 (4.6)		9 (3.8)	3 (1.9)	
Medicare + Private	66 (32.7)	59 (30.4)		75 (31.9)	50 (31.1)	
None	5 (2.5)	6 (3.1)		8 (3.4)	3 (1.9)	
Other (+gvt)	12 (5.9)	12 (6.2)		17 (7.2)	7 (4.3)	
**Education**			0.760			0.589
Highschool equiv or less	63 (32.0)	66 (35.9)		76 (33.6)	53 (34.2)	
Some college	33 (16.8)	33 (17.9)		36 (15.9)	30 (19.4)	
Associate, Bachelor, or vocation	60 (30.5)	53 (28.8)		66 (29.2)	47 (30.3)	
Master or Doctorate	41 (20.8)	32 (17.4)		48 (21.2)	25 (16.1)	
**Income**			0.126			0.840
< $25,000	28 (13.9)	33 (17.6)		35 (15.3)	26 (16.3)	
$25,000 to < $50,000	40 (19.9)	32 (17.0)		45 (19.7)	27 (16.9)	
$50,000 to < $75,000	21 (10.4)	16 (8.5)		22 (9.6)	15 (9.4)	
$75,000 or more	45 (22.4)	27 (14.4)		45 (19.7)	27 (16.9)	
Unknown	67 (33.3)	80 (42.6)		82 (35.8)	65 (40.6)	
**Marital Status**			0.188			0.054
Married	121 (59.9)	103 (53.1)		123 (52.6)	101 (62.3)	
Divorced/widow/separated/Single	81 (40.1)	91 (46.9)		111 (47.4)	61 (37.7)	
